# 2018 New Year Address of *Zoological Research*

**DOI:** 10.24272/j.issn.2095-8137.2018.011

**Published:** 2018-02-09

**Authors:** Yong-Gang Yao, Xue-Long Jiang

**Affiliations:** 1 Kunming Institute of Zoology, Chinese Academy of Sciences, Kunming 650223, China

At the beginning of a wonderful new year, we look back at *Zoological Research* (*ZR*) in 2017. We are very grateful to all our readers and authors for your dedication and continued support of *ZR*. Your ideas, input, and enthusiasm have been of immense value in helping us to improve the journal. Here, we would like to share a few memorable events and people of the past year. 

Firstly, it is with deep regret and sorrow that we note the passing of Colin Peter Groves, Professor of Biological Anthropology from the Australian National University in Canberra, Australia, on 30 November 2017. He was a long-standing and loyal friend of *ZR*, and joined our editorial board in 2014. He fulfilled his duties with dedication and diligence, and shared his outstanding expertise with opinions and constructive suggestions on many submissions to the journal. Despite his poor health, he continued to contribute to the journal (Groves, 2016) and was a respected member of our editorial board. We greatly cherish his contributions and generosity. 

We have also been inspired in the past year by the continued improvement in academic quality, total citations, and general influence of each of our publications. *ZR* achieved an estimated impact factor of 0.73 (based on the citation data from the Web of Science) and CiteScore of 0.84 (dated 11 January 2018, Scopus). We have also been fortunate with the addition of eleven outstanding experts who recently joined *ZR* as editorial members, including Yu-Hai Bi (Institute of Microbiology, Chinese Academy of Sciences (CAS), China), Peng-Fei Fan (Sun Yat-Sen University, China), Patrick Giraudoux (University of Franche-Comté, France), Cyril C. Grueter (University of Western Australia, Australia), Wei-Zhi Ji (Kunming Institute of Zoology, CAS, China), Shu-Qiang Li (Institute of Zoology, CAS, China), Wen-Jun Liu (Institute of Microbiology, CAS, China), Julian Kerbis Peterhans (Roosevelt University, USA), Xiang-Guo Qiu (University of Manitoba, Canada), Rui-Chang Quan (Xishuangbanna Tropical Botanical Garden, CAS, China), and Christian Roos (Leibniz-Institute for Primate Research, Germany). These remarkable specialists will bring new ideas and expertise to help reshape the journal. We are confident that our excellent editorial board will further facilitate the pivotal and active role of *ZR* in the field of science and publishing. 

In 2017, the “Project for Enhancing International Impact of China STM Journals” (PIIJ) (Class B) (2016–2018), the most extensive and influential journal fostering project in China, continued its support of *ZR*. With great effort from all editors and staff from the editorial office, *ZR* has been ranked among the top 300 “Outstanding S&T Journals of China”. Moreover, with the successive release of special issues focusing on academic topics of current interest, *ZR* has evolved into a vibrant journal with appreciable readability. 

In February 2017, *ZR* successfully hosted the “2017 Frontiers in Zoology Symposium” with the theme of “Animal Genomics and Ecological Protection”. We hope to replicate this success with the “2018 Frontiers in Zoology Symposium”, which will focus on “Comprehensive Scientific Investigation of Animals on the Tibetan Plateau and East Asia” (please find details at www.zoores.ac.cn). As such, we hope to see you in Kunming this coming spring. *ZR* also co-hosted the 2017 Annual Conference of the Chinese Herpetology Society. Such events are wonderful ways in which to meet up the old friends and colleagues as well as establish new contacts and share achievements and opinions.

To facilitate scientific communication and promote subject development, *ZR* is not only increasing its academic value, but also consistently reinforcing the ethical integrity accompanying academic publications. In 2016, *ZR* published an editorial (Liu, 2016), letter to the editor (Joob & Wiwanitkit, 2016), and statement (http://www.zoores.ac.cn/EN/abstract/abstract3772.shtml) regarding proper authorship. Publishing is of importance in almost every stage of a researcher’s career (Editorial Office of Zoological Research, 2016). Nowadays, under the background of big science, the advancement and elucidation of scientific questions often require collaboration among independent research groups, even those from different fields, to combine their expertise or specialties. Academia benefits from such extensive collaborations. As a result, we have observed that the proportion of co-first or co-corresponding author articles has significantly increased during the last few decades in many scientific journals. Akhabue & Lautenbach (2010) studied original research articles with equally contributing authors among five top life science journals, i.e., *New England Journal of Medicine*, *Journal of American Medical Association*, *Annals of Internal Medicine*, *Lancet*, and *British Medical Journal*. In 2000, the proportions of co-first or co-corresponding author papers in the five journals were <1%, 0%, 0%, <1%, and 0%, whereas in 2009, the proportions had significantly increased to 4.4%, 2.3%, 1.6%, 2.7%, and <1%, respectively. Li et al. (2013) studied the numbers of equally contributing articles in three major anesthesia journals (*Anaesthesia*, *British Journal of Anaesthesia*, and *Anesthesia & Analgesia*) over a 10-year period, and found that such papers increased significantly from 2002 to 2011 (0.9% to 8.8%, 0% to 8.8%, and 0.3% to 3.4%, respectively). The *Journal of Genetics & Genomics* published 124 and 85 original articles in 2007 and 2016, respectively, with co-first or co-corresponding author papers accounting for 16% (20/124) and 41.1% (35/85), respectively. In *Cell Stem Cell*, the percentage of co-first and co-corresponding author articles increased from 20.5% (18/88) in 2007 to 37.6% (77/205) in 2016. This trend reinforces collaborations and data sharing. For *ZR*, in 2007, approximately 9% (9/108) of the original research articles published were by two corresponding or co-first authors; by 2016, this increased to 33% (12/36). 

For most academic journals there are no universal rules regarding shared authorship, with different journals usually having their own specific regulations. For example, the *American Journal of Human Genetics* states that “unrestricted joint authorship is allowed” and “a maximum of two corresponding authors is allowed” (http://www.cell.com/ajhg/authors). *Neuron* merely restricts the number of lead contacts (i.e., the corresponding author responsible for communicating with the journal and helping facilitate other issues regarding submission) to only one, but not other authors (http://www.cell.com/neuron/authors#policies). The *Journal of Neurosciences* does not restrict co-authors numbers, but states “honorary authorship” to be a “misrepresentation” (http://www.sfn.org/member-center/professional-conduct/guidelines-for-responsible-conduct-regarding-scientific-communication#menulevel1). *Scientific Reports* allows up to six co-authors, with corresponding authors usually limited to three.

*ZR* has an explicit attitude and firm stance to proper authorship and resolutely complies with the criteria of the International Committee of Medical Journal Editors (International Committee of Medical Journal Editors, 2013). Accordingly, *ZR* would consider guest/ghost authors unethical. *ZR* appreciates and encourages positive and comprehensive collaboration. Combining findings from different researchers can yield a more complete story, and emphasizing equal contribution of involved parties is fair and encourages team work and collaborations. However, it is commonly accepted, at least in the biomedical field, that publication credit can never be enjoyed equally among all listed authors in the byline, with the first and last (corresponding) authors usually receiving most recognition, unless shared authors are clearly listed in alphabetical order. The addition of equally contributing authors can dilute the credit allocated to each author. These actions obviously deviate from the original intention for properly sharing credit. 

Although *ZR* encourages every author of a paper to read the authorship guidelines thoroughly and carefully before submission, we have not previously insisted upon an author-contribution statement. However, we believe it is important to increase transparency and enhance authorship integrity. Thus, for submissions to *ZR* after 1 January 2018, an author-contribution statement will be compulsory. The statement is not required in uniform format, but the contribution of every person in the author list must be specifically described. Moreover, for collaborative studies, *ZR* requires that every person involved in the paper agrees with the author list and sharing of the credit. 

Whether these measures will make a substantial difference in eliminating unethical authorship remains to be seen. However, we believe such ambitions are worth attempting simply because transparency and fairness are always worthy of pursuit. With your support and positive contributions, *ZR* will remain a respectable platform for scientific communication.

Again, thank you all. May your new year hold all the excitement, promise, and good wishes we have shared in 2017.

Sincerely,


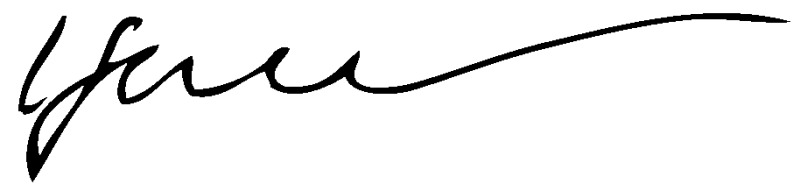

Yong-Gang Yao, Editor-in-Chief
Kunming Institute of Zoology,
Chinese Academy of Sciences, Kunming Yunnan 650223, China 
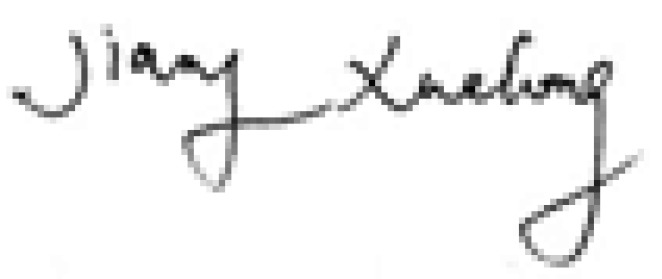

Xue-Long Jiang, Executive Editor-in-Chief
Kunming Institute of Zoology,
Chinese Academy of Sciences, Kunming Yunnan 650223, China

